# Surgical and Quality of Life Outcomes Following Robotic-Assisted (da Vinci) Laparoscopic Repair of Vesicovaginal Fistula: A Case Report and Video Demonstration

**DOI:** 10.7759/cureus.42171

**Published:** 2023-07-19

**Authors:** Elias Tsakos, Emmanouil M Xydias, Apostolos C Ziogas, Kanelina Bimpa, Stergios K Doumouchtsis, Georgios Karydas, Vasileios Moros, Vasileios Poulakis

**Affiliations:** 1 Department of Obstetrics and Gynaecology, EmbryoClinic IVF, Thessaloniki, GRC; 2 Department of Robotic Gynaecological Surgery, St. Luke's Hospital, Thessaloniki, GRC; 3 Department of Medicine, University of Thessaly, School of Health Sciences, Larissa, GRC; 4 Department of Breast Surgery, EmbryoClinic IVF, Thessaloniki, GRC; 5 Department of Obstetrics and Gynaecology, Epsom and St. Helier University Hospitals, Epsom, GBR; 6 Department of Urology, St. Luke's Hospital, Thessaloniki, GRC; 7 Department of Urology, Metropolitan Hospital, Athens, GRC

**Keywords:** urogynaecology, quality of life (qol), vesicovaginal fistula, da vinci robotic system, robotic-assisted laparoscopic gynecologic surgery, ai & robotics healthcare

## Abstract

This report presents the case of a 63-year-old woman who developed a vesicovaginal fistula as a complication of a previous total hysterectomy. The fistula was treated with the use of the da Vinci X surgical system by a multi-disciplinary operating team, including senior Robotic Urological and Gynecological Surgeons at St. Luke’s Hospital in Thessaloniki, Greece. The patient was monitored up to 12 months post-op at the time of writing and she was asked to evaluate post-op quality of life using the SF-36 and ICIQ-SF-UI questionnaires. The robotic surgical procedure was completed successfully. The total operation duration was 105 minutes, without any intra-operative complications. The patient was hospitalized for two days and made a swift, uneventful recovery. Regarding the quality of life, the patient reported satisfactory improvement in almost every domain assessed compared to her pre-op assessment; an improvement that was maintained throughout the reported follow-up period. At the time of writing, the patient reports no long-term complications and satisfactory urinary continence. Robotic-assisted laparoscopic vesicovaginal fistula repair is an effective and safe treatment option for this rare complication, as indicated by both post-operative data and the patient’s own self-evaluation in this report. Further research is warranted, focusing on refining the surgical technique and comparing this to other alternative methods aiming to further improve patient outcomes.

## Introduction

Vesicovaginal fistula (VVF) is a pathologic connection between the bladder and the vagina via a patent, tubal tract, resulting in continuous urinary incontinence (UI) [1,2]. The condition has been known since antiquity [3] and remains prevalent today. It is estimated to affect up to 3 million women and rates may be as high as 130,000 new cases per year in developing countries [4,5], due to the increased rates of obstetric complications and prolonged obstructed labor in particular, which constitute the primary etiology for VVF in these regions [5,6]. In developed countries, VVF is most commonly a complication of pelvic or gynecological surgery [7] and sometimes of radiotherapy, trauma, or malignancy in the pelvis and thus significantly more rare, compared to the cases resulting from obstetric complications [8].

Clinical suspicion of VVF should arise in cases of severe UI manifesting a few weeks after pelvic surgery, rarely within multiple weeks or months [1]. Conservative treatments via catheterization and continuous urine drainage have been proposed, optionally with adjunct oral antimuscarinics and antibiotics; with more advanced techniques, such as cauterization, fibrin glue or platelet-rich plasma injections also having been tested [1,9]. However, the majority of cases are treated surgically, via abdominal, vaginal, or laparoscopic VVF repair, with the vaginal approach being preferred, due to superior success rates [7] and other practical advantages [10].

In recent years, the robotic-assisted surgical approach has gained ground and has earned the recommendation of the European Association of Urology [11]. Given the complexity of the procedure and the significant impact on patients' quality of life, detailed descriptions of the robotic repair technique are imperative, along with an assessment of clinical and quality of life improvement. The aim of the present case report is to present a case of VVF repair via the Robotic-assisted laparoscopic (da Vinci X) approach, to provide a video demonstration of the applied technique, and to assess its impact on patient quality of life.

## Case presentation

A 63-year-old woman, para 2, gravida 2, overweight (BMI: 29.7), visited our clinic, reporting severe urine incontinence (UI) two and a half weeks after gynecological surgery. Her relevant personal history started when she first visited the clinic reporting menstrual irregularities, which had begun 13 years prior and kept worsening, leading to a clinical suspicion of a fibroid uterus. Two and a half weeks prior to her visit due to UI, she underwent a total robotic-assisted (da Vinci) hysterectomy, with bilateral salpingo-oophorectomy.

**Figure 1:**
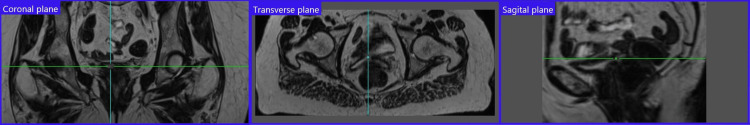
Multi-planar view of the vesicovaginal fistula VVF in MRI scan. VVF: vesicovaginal fistula, MRI: Magnetic Resonance Imaging

Eighteen days post-operatively, the patient reported severe UI, with total loss of bladder control and severe impact on quality of life. She was instantly diagnosed with VVF at an outpatient clinic, a diagnosis later confirmed via MRI (Figure 1) and cystoscopy. Initial management entailed the insertion of ureteral catheters (“pigtail” type) bilaterally and a urethral catheter, all of which were kept in place for three months. This intervention aimed at allowing the patient to return to her daily routine, while simultaneously facilitating VVF healing without surgical intervention. However, UI persisted and eventually, the patient opted for surgical treatment, four months following her initial pelvic surgery.

The technique used has been described in detail elsewhere [12] by Dr. VP, who was also the lead surgeon among the surgical team in this case. The operation was conducted using the da Vinci X Surgical System®, with the following surgical instruments: fenestrated bipolar forceps, Maryland bipolar grasper, monopolar curved scissors and needle holder. Under general anesthesia, the patient was placed in lithotomy position and via cystoscopy (22Fr cystoscope), two J stents 7Fr were inserted under radioscopic guidance, exiting through the urethra. A 6 Fr ureteral catheter was placed through the bladder orifice of the VVF and pulled through the vaginal orifice, resulting in one end exiting through the urethra and the other through the vagina, mapping the fistulous tract. Subsequently, a urethral 16 Fr Foley catheter was inserted, and the patient was switched to a supine, mild Trendelenburg position, with the patient’s legs in mild flexion and abduction.

**Figure 2:**
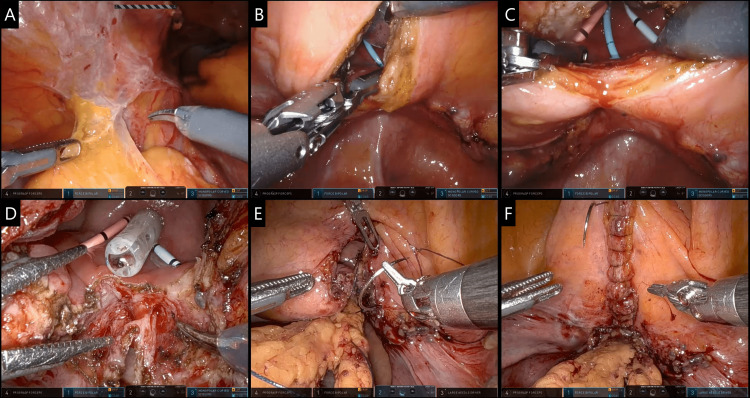
Robotic-assisted laparoscopic VVF repair. (A) Adhesiolysis and omental mobilization, (B) cystectomy at the posterior bladder wall, (C) detection of VVF (blue catheter), protection of right (light blue catheter), and left (red catheter) ureters, (D) scoring and resection of the fistulous tract, dissection and separation of anterior bladder aspect and vagina, (E) suturing of vagina and interposition of omental pedicle between it and the bladder, and (F) suturing of bladder incision and subsequent intra-peritoneal drain placement.

Four robotic trocars (8mm) were placed for the console surgeon and two more for the assistant (12 mm and 5 mm). Central docking and right-side assistant configuration were used. The operation was initiated with adhesiolysis and mobilization of the sigmoid and cecum. A 3-cm vertical incision was made at the posterior wall of the bladder, the VVF opening was located, and its catheter was removed. The VVF was circularly scored with the monopolar scissors. The bladder was meticulously dissected and detached from the vagina, and both were debrided. The vaginal incision was sutured with continuous V-loc® 2.0 sutures. The omentum was mobilized down to the pelvis and an omental pedicle was inter-positioned between the bladder and the vagina and anchored in place via Vicryl 2.0 sutures, aiming at facilitating healing and preventing recurrence. The bladder incision was sutured using V-loc® 2.0 sutures and bladder water-tightness was confirmed via 120 mL saline infusion. Hemostasis check was performed and an intra-peritoneal Blake® multi-lumen silicon drain was inserted in the lesser pelvis through a leftover trocar opening. Skin closure was performed with surgical clips and the patient was uneventfully extubated. Surgical procedure highlights are depicted in Figures 2A-2F, with a thorough step-by-step description being available in video form (Video 1).

**Video 1:** Edited, step-by-step video demonstration of the robotically assisted VVF repair procedure.

Overall surgical time was 105 minutes, with a console time of 55 minutes. Estimated blood loss was 100 mL and no blood transfusion was required. No post-operative complications were noted, and the patient made a full and timely recovery. For one month post-operatively, a urethral (Folley’s) catheter was kept in place aiming at facilitating healing and following that, except for a mild urinary tract infection treated successfully with a single course of oral antibiotics, no other late complications were reported whatsoever.

**Figure 3:**
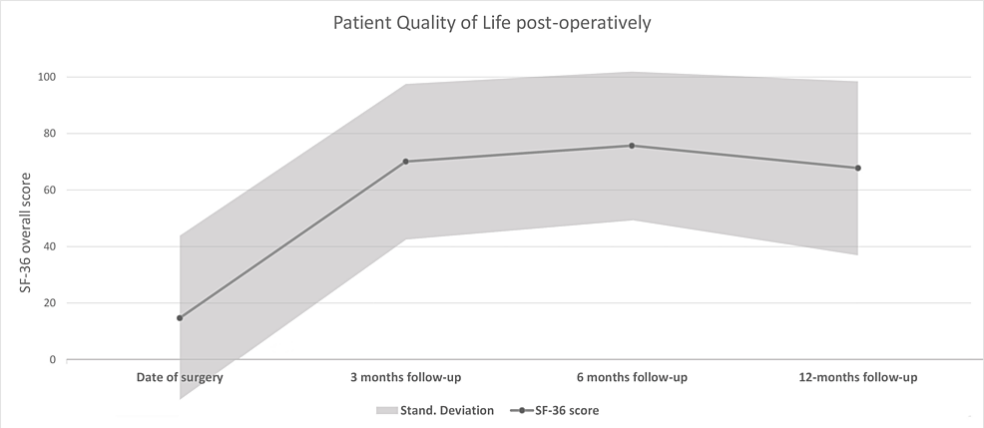
Line plot of Short Form 36 (SF-36) questionnaire score at surgery and at subsequent follow-ups. Statistically significant improvement in patient quality of life, maintained up to one year post-operatively.

In order to more accurately assess the treatment’s impact on the patient’s quality of life, the MOS 36-Item Health Survey [13] was used for an overall assessment of physical, psychological and social well-being and the International Consultation on Incontinence Questionnaire-Urinary Incontinence Short Form (ICIQ-UI SF) [14] for UI symptoms in particular. These questionnaires were completed initially two weeks prior to surgery and subsequently at the three-, six-, and 12-month follow-ups, so as they could be examined comparatively. Results showed that there was statistically significant improvement in overall SF-36 score compared to the pre-op condition, which was maintained throughout the follow-up period (Figure 3).

There was significant improvement in almost every SF-36 domain, with exceedingly notable changes in physical functioning, social functioning and pain in particular. ICIQ-UI SF scores also improved significantly, indicating a satisfactory degree of urinary continence (Table 1). Post-hoc analysis showed that the most significant improvement occurred between the pre-operative baseline status and any of the post-operative scores, while amongst post-operative scores, patient satisfaction was maintained throughout the first post-operative year (Table 2).

**Table 1:** Results of quality-of-life assessment. Short form 36 (SF-36) and International Consultation on Incontinence Questionnaire–Urinary Incontinence Short Form (ICIQ-UI SF) questionnaire mean scores at surgery and at different follow-up assessments, per domain. Means are compared using the one-way analysis of variance (ANOVA) test.

	Domains	Score prior to surgery, Mean ±SD	Score at 3 months follow-up, Mean ±SD	Score at 6 months follow-up, Mean ±SD	Score at 12 months follow-up, Mean ±SD	F-value	P-value
SF-36 Questionnaire	Physical functioning	0±0	75±25	80±24.49	85±22.91	36.96	*P < 0.001
	Role limitations due to physical health	0±0	75±43.3	100±0	75±43.3	8.001	*P = 0.003
	Role limitations due to emotional problems	33.3±47.14	66.7±47.14	66.7±47.14	33.3±47.14	0.502	P = 0.691
	Energy/fatigue	25±8.66	65±16.58	65±29.58	65±16.6	4.265	*P = 0.029
	Emotional well-being	24±23.32	72±9.8	72±20.4	60±25.3	6.085	*P = 0.006
	Social functioning	0±0	62.5±12.5	75±0	62.5±12.5	10.705	*P = 0.022
	Pain	10±10	77.5±2.5	77.5±2.5	77.5±2.5	76.737	*P < 0.001
	General health	35±37.42	65±25.5	65±20	50±0	1.683	P = 0.212
	Overall	14.71±29.14	70.12±27.45	75.72±26.21	67.8±30.7	36.053	*P < 0.001
ICIQ-UI Short Form	UI during the past month	20 (very severe)	1 (slight)	2 (slight)	4 (slight)	Significant improvement	

**Table 2:** Results of post-hoc analysis. Tukey’s post-hoc analysis tests, comparing mean Short form 36 (SF-36) scores in pairs of follow-up assessments, per domain.

	Domains	Pre-op versus 3 month post-op score	Pre-op versus 6 month post-op score	Pre-op versus 12 month post-op score	3 months post-op versus 6 months post-op	3 months post-op versus 12 months post-op	6 months post-op versus 12 months post-op
SF-36 Questionnaire	Physical functioning	Diff.=75, 95%CI: 49.8-100.2, P<0.001*	Diff.=80, 95%CI: 54.8-105.2, P<0.001*	Diff.=85, 95%CI: 59.8-110.2, P<0.001*	Diff.=5, 95%CI: -20.2-30.2, P=0.95	Diff.=10, 95%CI: -15.2-35.2, P=0.7102	Diff.=5, 95%CI: -20.2-30.2, P=0.95
	Role limitations due to physical health	Diff.=75, 95%CI: 10.72-139.28, P=0.00211*	Diff.=100, 95%CI: 35.72-164.27, P=0.0029	Diff.=75, 95%CI: 10.72-139.28, P=0.00211*	Diff.=25, 95%CI: -39.3-89.3, P=0.6647	Diff.=0, 95%CI: -64.3-64.3, P=1	Diff.=25, 95%CI: -89.3-39.3, P=0.6647
	Role limitations due to emotional problems	Diff.=33.4, 95%CI: -89.8-156.6, P=0.8212	Diff.=33.4, 95%CI: -89.8-156.6, P=0.8212	Diff.=0, 95%CI: -123.3-123.3, P=1	Diff.=0, 95%CI: -123.3-123.3, P=1	Diff.=33.4, 95%CI: -156.6-89.8, P=0.8212	Diff.=33.4, 95%CI: -156.6-89.8, P=0.8212
	Energy/ fatigue	Diff.=40, 95%CI: 0.108-79.89, P=0.0494*	Diff.=40, 95%CI: 0.108-79.89, P=0.0494*	Diff.=40, 95%CI: 0.108-79.89, P=0.0494*	Diff.=0, 95%CI: -40.7-40.7, P=1	Diff.=0, 95%CI: -40.7-40.7, P=1	Diff.=0, 95%CI: -40.7-40.7, P=1
	Emotional well-being	Diff.=48, 95%CI: 10.74-85.26, P=0.0097*	Diff.=48, 95%CI: 10.74-85.26, P=0.0097*	Diff.=36, 95%CI: -1.26-73.3, P=0.0601	Diff.=0, 95%CI: -37.3-37.3, P=1	Diff.=-12, 95%CI: -49.3-25.3, P=0.7939	Diff.=-12, 95%CI: -49.3-25.3, P=0.7939
	Social functioning	Diff.=62.5, 95%CI: 2.94-122.1, P=0.0428*	Diff.=75, 95%CI: 15.44-134.56, P=0.0231*	Diff.=62.5, 95%CI: 2.94-122.1, P=0.0428*	Diff.=12.5, 95%CI: -47.06-72.06, P=0.8277	Diff.=0, 95%CI: -59.56-59.56, P=1	Diff.=12.5, 95%CI: -47.06-72.06, P=0.8277
	Pain	Diff.=67.5, 95%CI: 45.32-89.68, P<0.001*	Diff.=67.5, 95%CI: 45.32-89.68, P<0.001*	Diff.=67.5, 95%CI: 45.32-89.68, P<0.001*	Diff.=0, 95%CI: -22.2-22.2, P=1	Diff.=0, 95%CI: -22.2-22.2, P=1	Diff.=0, 95%CI: -22.2-22.2, P=1
	General health	Diff.=30, 95%CI: -14.8-74.8, P=0.2603	Diff.=30, 95%CI: -14.8-74.8, P=0.2603	Diff.=15, 95%CI: -29.8-59.8, P=0.7744	Diff.=0, 95%CI: -44.8-44.8, P=1	Diff.=15, 95%CI: -29.8-59.8, P=0.7744	Diff.=15, 95%CI: -29.8-59.8, P=0.7744
	Overall	Diff.=55.41, 95%CI: 38-72.83, P<0.001*	Diff.=61.01, 95%CI: 43.6-78.43, P<0.001*	Diff.=53.09, 95%CI: 35.67-70.51, P<0.001*	Diff.=5.6, 95%CI: -11.82-23.02, P=0.8373	Diff.=-2.32, 95%CI: -19.74-15.01, P=0.9857	Diff.=-7.92, 95%CI: -25.34-9.5, P=0.63.91
ICIQ-UI Short Form	UI during the past month	Significant improvement	Significant improvement	Significant improvement	Stable condition	Stable condition	Stable condition

## Discussion

In this report, we present a case of vesico-vaginal fistula, originating as a surgical complication, and its repair via the robotic-assisted laparoscopic approach with the use of the da Vinci X Surgical System®. Overall, the applied technique demonstrated significant surgical efficacy, completely restoring urinary continence, combined with overall safety, negligible blood loss, swift and complete recovery, and the absence of any short- or long-term complications. This assessment has been additionally corroborated by the patient’s own perspectives; there was a significant improvement in UI-specific, as well as general, quality of life. This improvement was maintained throughout the reported follow-up period of 12+ months to date.

The formation of a VVF is a very distressing complication of obstetrical and gynecological procedures, which may severely impact patient quality of life, with major physical, psychological, and social impairment [15,16]. Optimal treatment of VVF is still debated, with multiple treatment approaches proposed in the scientific literature. In a recent study, conservative treatment has been shown to be successful in 92.86% (95% CI: 79.54-99.89) of cases, whereas surgical treatment was successful in 97.98% (95% CI: 96.13-99.29) of cases; the latter being by far the most popular treatment option and being applied to 96.4% of all participants [16]. Regarding surgical treatment options, the authors reported that the abdominal/trans-vesical approach had a success rate of 97.05% (95% CI: 94.55-99.18), the vaginal approach 93.82% (95% CI: 89.96-97.49), the laparoscopic/robotic-assisted approach 98.87% (95% CI: 96.85-99.99) and a combined transvaginal-transabdominal approach 90.70% (95% CI: 64.63-99.87) [16].

Robotic-assisted surgery has been widely and successfully applied in the treatment of several benign gynecological conditions, such as endometriosis [17], uterine fibroids [18], uterine prolapse [19], and benign hysterectomy [20]. With regard to VVF robotic-assisted treatment in particular, Bodner-Adler et al. [16], similarly to the present case report, reported a 100% success rate, however, due to the limited number of studies and patients, no concrete scientific conclusions could be extracted. Another study comparing robotic and abdominal approaches concluded that, while there was no statistically significant difference regarding successful management, there was a difference in blood loss (88 vs. 170 mL, p < 0.05) and in mean hospitalization duration (mean 3.1 vs. 5.6 days) [21], favoring the robotic approach. These trends were consistent with the observations made in the present study; our patient experienced negligible blood loss and was hospitalized for two days only.

Chandna et al. [22], using a similar technique to the one described in the present report, reported a success rate of 93.9%, a mean operative time of 165.3 ± 52.4 minutes with negligible blood loss (66.8 ± 44.2 mL). Overall, the authors concluded that the robotic approach is a safe and effective treatment option for this complex pathology, achieving satisfactory results with negligible complications [22]. Kidd et al. [23] conducted a retrospective study and observed that robotic-assisted repair had a median operating time of 187 minutes (IQR: 151-219), minimal blood loss (median 50 mL, IQR: 50-93), and a median hospitalization time of one day (IQR: 1-2), with only two patients (9%) having a postoperative complication. Eventually, 20 out of 22 included patients (91%) were free of UI at follow-up [23].

The present report, to the best of our knowledge, is the first describing a case of robotic-assisted VVF repair from a multi-disciplinary surgical team in Greece. The general trends identified in the available literature were consistent with our observations during the management of this case; namely that robotic-assisted repair of VVF, using the technique described, offers an optimal balance between effectiveness and safety. The main strength of this report is the detailed description of the surgical procedure, accurately demonstrated in the accompanying video, which has been edited to provide a short, but detailed, educational, step-by-step description of the procedure. An additional strength of this case report is the inclusion of the patient’s perspective throughout the first post-operative year via quality-of-life assessment questionnaires. These data provide valuable insight into the effect of the intervention on overall quality of life, which is particularly important when treating UI, a manifestation that severely affects all aspects of a patients’ life.

## Conclusions

Robotic-assisted laparoscopic repair is an effective and safe treatment option for VVF, as proven by both post-operative data and the patient’s own self-evaluation in this report. Further research should focus on more long-term follow-up of patients following robotic-assisted VVF repair, in order to examine recurrence and long-term complication rates of this technique; a course of action that will be applied on the present case as well. Additionally, researchers may focus on refining the surgical technique and on comparing it to alternatives aiming at further improvement of surgical and quality of life outcomes.
